# Phytochemical studies on traditional herbal medicines based on the ethnopharmacological information obtained by field studies

**DOI:** 10.1007/s11418-021-01545-7

**Published:** 2021-07-13

**Authors:** Naonobu Tanaka, Yoshiki Kashiwada

**Affiliations:** Graduate School of Pharmaceutical Sciences, Tokushima University, Tokushima, 770-8505 Japan

**Keywords:** Phytochemical study, Field study, Traditional herbal medicine, Ethnopharmacological information

## Abstract

Traditional herbal medicines, which have been used in the matured traditional medical systems as well as those have been used in ethnic medical systems, are invaluable resources of drug seeds. Ethnobotanical and ethnopharmacological survey may provide useful information of these herbal medicines, which are valuable for searching new bioactive molecules. From this viewpoint, we have been performing the ethnobotanical and ethnopharmacological field studies in Yunnan Province and Guangxi Zhuang Autonomous Region, China, and Mongolia. Phytochemical studies on traditional herbal medicines were performed based on the information obtained by our ethnobotanical survey. Herbal medicines used in Uzbekistan and Bangladesh were also investigated on the basis of the ethnopharmacological information obtained from collaborative researchers in the respective regions. Some studies were carried out for searching active substance(s) based on bioassay-guided fractionation and isolation. Over 150 new molecules were isolated in these studies, and their various biological activities were also demonstrated. This review summarizes the results of phytochemical studies of those traditional herbal medicines as well as biological activities of the isolated molecules.

**Graphic abstract:**

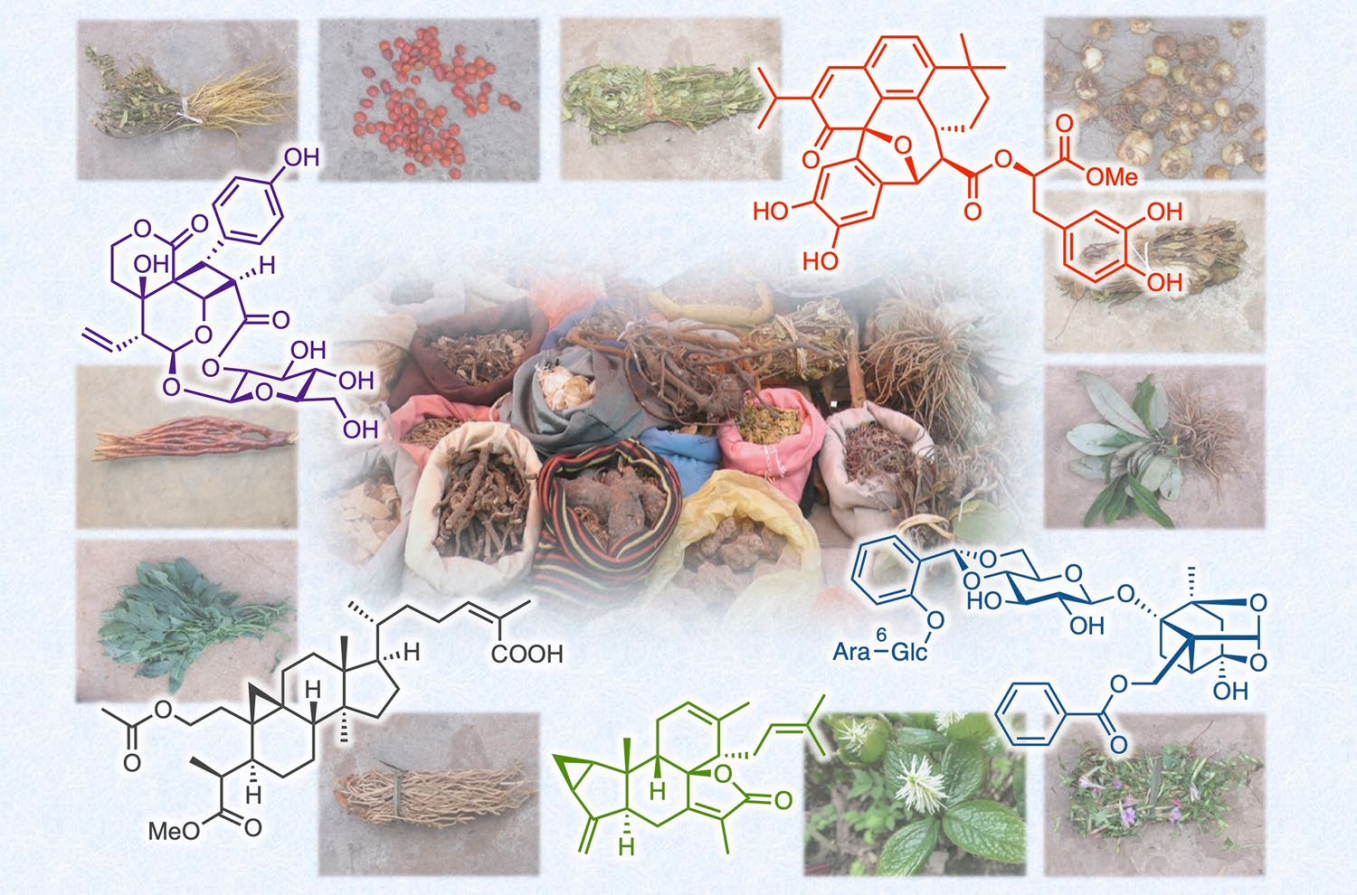

## Introduction

A variety of herbal medicines have been utilized in traditional medical systems as well as ethnic populations in various areas of the world. Natural products, particularly unique secondary metabolites found from those herbal medicines, including those have been used in not only traditional medical systems but also ethnic medical systems, provide potential molecules to develop new drug candidates. Therefore, these herbal medicines are considered to be invaluable resources of drug seeds molecules on which new therapeutic agents can be based. Chinese, Ayurvedic, and Unani are matured traditional medical systems, where several knowledges of herbal medicines have been documented. In contrast, some ethnic medical systems employ unique uses of herbal medicines or those prepared from endemic resources in respective regions. In addition, some of those knowledges have been preserved through the ages, in some case, simply in orally, and those remained only in isolated ethnic groups [1]. Ethnobotany and ethnopharmacology, a kind of scientific field study for interrelations between ethnic people and herbal medicines, may provide undocumented unique information of herbal medicines leading ideas of research to find sources of new molecules [2]. From this viewpoint, we have been collaborating with researchers in several countries, of which some have been performing ethnobotanical and ethnopharmacological surveys with the researchers based on the academic agreements between universities, while the others are collaborative studies with the researchers based on the exchange of ethnopharmacological information. In this review, research progress of our phytochemical studies on traditional herbal medicines used in China, Mongolia, Uzbekistan, and Bangladesh will be described.

## Geographical and ethnobotanical features of Yunnan Province and Guangxi Zhuang Autonomous Region, China

China has 55 minority groups, recognized officially by the government, that settled in particular areas and have been using a variety of ethnic herbal medicines as their own remedies different from the matured Chinese traditional medical system. Yunnan Province and Guangxi Zhuang Autonomous Region are the areas, where many ethnic minority groups are living. Yunnan, an inland province at a low latitude in southwestern China, has a characteristic geographical feature with the large height difference; the lowest point of 76.4 m above sea level at the Red River at the southeast edge, and the highest point of 6740 m at the summit of Kagebo peak. The climate of Yunnan Province is also affected by geographic location neighboring tropical and subtropical regions. For this reason, Yunnan has a vast diversified and unique natural resources, where more than 18,000 higher plant species are distributed [3]. In contrast, limestone areas of Guangxi Zhang Autonomous Region cover up to 40% areas of this province, which comprises one of the largest limestone areas of the world if considering to combine with limestone areas of neighboring Guizhou and Yunnan provinces, and North Vietnam. A diverse flora of over 8000 vascular plants and very high levels of endemism, in which 10 genera and 744 species, are endemic to this area [4].

## Phytochemical studies on traditional herbal medicines used in Yunnan Province

Twenty-five ethnic minority groups, out of 55 recognized officially by the Chinese government, live in their own autonomous areas in Yunnan Province, and those minority groups have long used many of diverse plants grown in Yunnan as their own unique herbal medicines. Our group performed an ethnopharmacological survey collaborated with researchers of the Kunming Institute of Botany, Chinese of Academy of Sciences, and some phytochemical studies based on the information obtained in the ethnopharmacological survey.

The genus *Gentiana*, the largest member of Gentianaceae, consists of about 400 species. Various *Gentiana* plants have been included in herbal remedies for poor appetite and digestive problems worldwide [5]. *G. rigescens* Franch. ex Hemsl. grows in southwestern part of China, especially in the mountain areas of Yunnan Province. The roots of *G. rigescens*, one of the varieties of Gentianae Radix (Long-Dan) in Chinese Pharmacopeia, have been commonly used as a traditional Chinese medicine, for the treatment of inflammation, and for hepatitis, rheumatism, and cholecystitis [6]. It also has been used for the treatment of hepatitis and cholecystitis by the Yi ethnic minority group living in Yunnan Province.

We conducted the phytochemical investigation on the aerial parts of *G. rigescens*. Thus, the MeOH extract of this plant material was partitioned between EtOAc and H_2_O, and the EtOAc-soluble fraction was further partitioned between *n*-hexane and 90% MeOH aq. The 50% MeOH aq.-soluble materials, obtained by subsequent partition of the 90% MeOH aq.-soluble materials with CHCl_3_ and 50% MeOH aq., were separated by chromatographies repeatedly to isolate seven new acylated secoiridoid glucosides, a new secoiridoid, and three new noriridoids as well as seven related known compounds including macrophylloside A. Their structures were assigned by detailed spectroscopic analyses and chemical evidence [7, 8].

**Fig. 1:**
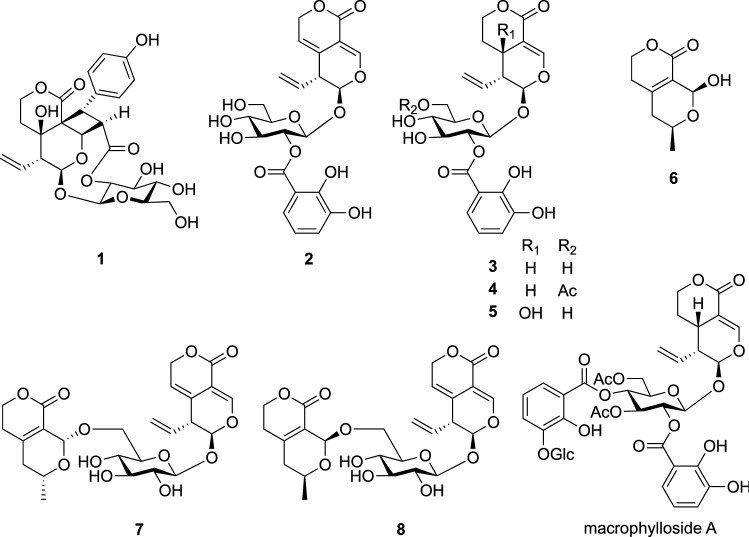
The structures of rigenolides A (**1**), B (**7**), C (**8**), F (**3**), G (**4**), and H (**5**), noriridoid (**6**), and macrophylloside A isolated from *Gentiana rigescens*

Among new acylated secoiridoid glucosides, rigenolide A (**1**) had a unique structure with a cyclobutane ring, being formed by intramolecular [2 + 2] cycloaddition between swertiamarine moiety and *p*-coumaroyl group bound to C-2′ of swertiamarine. This was the first example of a secoiridoid glucoside having a cyclobutane ring. Compound **6** was isolated as a partially racemic mixture. In contrast, repeated chromatographic separations of the H_2_O-soluble materials gave two new conjugates of norsecoiridoid and secoiridoid glucoside, rigenolides B (**7**) and C (**8**) [9]. The structures of **7** and **8** were elucidated on the basis spectroscopic analysis, chemical conversion, and TDDFT ECD calculation. Compound **6**, corresponding to the noriridoid moiety of **7** and **8**, might yield a pair of diastereomers corresponded to **7** and **8** by condensation with gentiopicroside. Macrophylloside A showed potent DPPH free-radical scavenging activity with an IC_50_ value of 16.2 μM, which was more potent than that of L-ascorbic acid used as a positive control. In contrast, **2**–**5**, possessing a 2,3-dihydroxybenzoyl moiety, showed moderate radical scavenging activities with IC_50_ values ranging from 36.4 to 48.2 μM, while **1** did not show a radical scavenging activity (Fig. 1).

**Fig. 2:**
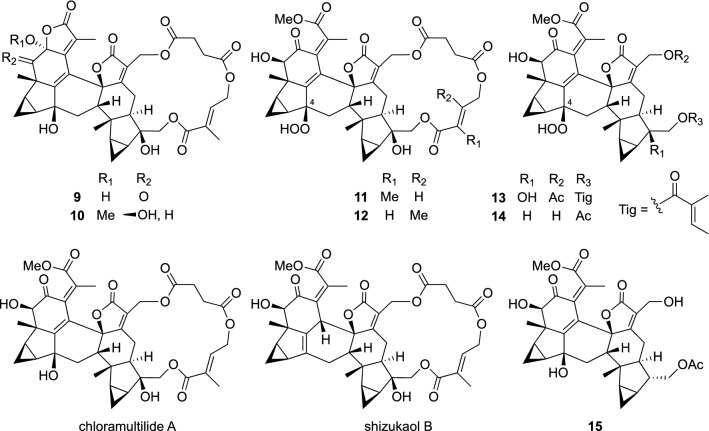
The structures of spicachlorantins A–G (**9**–**15**), chloramultilide A, and shizukaol B isolated from *Chloranthus spicatus*

Plants belonging to the genus *Chloranthus* (Chloranthaceae) are recognized as a rich source of sesquiterpenes, especially lindenane sesquiterpenes represent taxonomically characteristic secondary metabolites of the *Chloranthus* plants [10]. *C. spicatus* (Thunb.) Makino is an evergreen shrub native to southern China, and is also a horticultural plant widely cultivated in eastern Asia. We came to know in our ethnopharmacological study that the roots of *C. spicatus* have been used to treat bone fractures by the Va ethnic group, while the Dai ethnic group has been using this plant to treat high blood pressure. Chemical study on the roots of *C. spicatus* lead to the isolation of two new lindenane sesquiterpene dimers with an 18-membered macrocyclic ring, spicachlorantins A (**9**) and B (**10**), along with a known related compound, chloramultilide A (Fig. 2) [11]. The absolute configurations of **9** and **10** were elucidated by ECD spectroscopic analysis. Shizukaol B, a dimeric lindenane sesquiterpene possessing a macrocyclic ring, from *C. japonicus* was reported first in 1992 [12]. The related dimeric lindenane sesquiterpenes, shizukaols F, G, and H, from the same plant material were subsequently reported in 1995 [13]. Later on, further seven related dimeric lindenane sesquiterpenes from *C. multistachys*, *C. holostegius*, *C. henryi*, *C. tianmushanensis*, and *C. fortunei* were reported between 2006 and 2009 [14], and our report of the isolation of **9** and **10** from *C. spicatus* in 2009 followed those ones. Since the plant materials, *C. spicatus*, obtained in our survey in Yunnan were small, further investigation was carried out with cultivated plant materials, which resulted in the isolation of eight new lindenane sesquiterpene dimers, spicachlorantins C–J, along with seven known related sesquiterpene dimers. Spicachlorantins C–F (**11**–**14**) were the first examples of lindenane sesquiterpene dimers with a hydroperoxy group at C-4, and were considered to be biogenetic precursors for the corresponding hydroxy derivatives of dimeric lindenane sesquiterpenes distributed in *Chloranthus* plants. Spicachlorantin A (**9**) was also isolated from *C. angustifolius* by Yang et al., and its antifungal activity against *Candida albicans* (MIC 8 μg/mL) was reported [15]. In contrast, Wang et al. reported the isolation of spicachlorantins B (**10**) and G (**15**) from *C. henryi*, and demonstrated their anti-neuroinflammatory effect (IC_50_ values of 79.4 and 70.4 μM, respectively) by inhibiting nitric-oxide (NO) production in lipopolysaccharide (LPS)-stimulated murine BV-2 microglial cells with relatively low cytotoxicity [16].

**Fig. 3:**
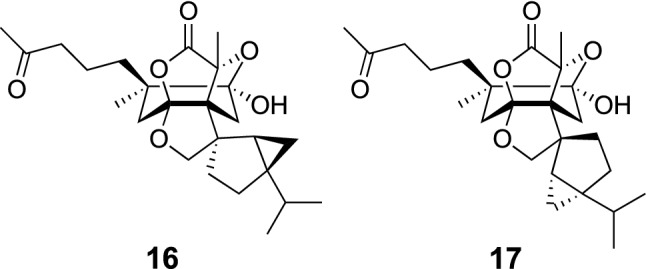
The structures of hitorins A (**16**) and B (**17**) isolated from *Chloranthus japonicus*

The findings of structurally unique secondary metabolites from *C. spicatus* prompted us to study on the constituents of *C. japonicus* as an extensive study of this research project. *C. japonicus* (hitorishizuka in Japanese) has been used as a herbal medicine by the Ainu people, an ethnic minority group living in Hokkaido, Japan, for the treatment of gastrointestinal disorders. This study resulted in the isolation of two novel C_25_ terpenoids with a 6/5/5/5/5/3 hexacyclic skeleton, hitorins A (**16**) and B (**17**) (Fig. 3) [17]. Hitorins A (**16**) and B (**17**) were considered to be biogenetically derived from eudesmane sesquiterpene and thujane monoterpene. Biomimetic synthesis of **16** and **17** has been reported by Li et al. as a preprinted manuscript in the ChemRxiv [18]. Evaluation of biological activities of **16** and **17** as well as further explorative study of the related terpenoids are underway.

**Fig. 4:**
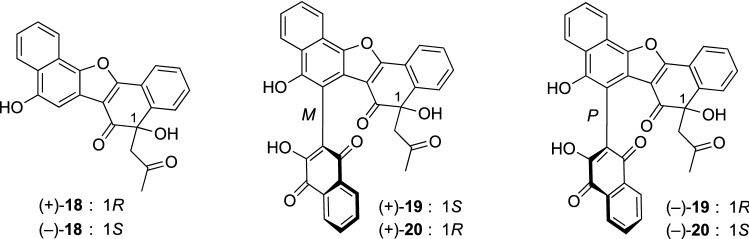
The structures of rubiaquinones A–C (**18**–**20**) isolated from *Rubia yunnanensis*

*Rubia yunnanensis* (Rubiaceae) is a perennial climbing herb distributed in mainland China. The roots of *R. yunnanensis* have been used as a herbal medicine to treat cancer, vertigo, insomnia, rheumatism, tuberculosis, menstrual disorders, and contusions [19]. Three new naphthoquinones, rubiaquinones A–C (**18**–**20**), were isolated from the 70% acetone aq. extract of the *R. yunnanensis* roots. Rubiaquinone A (**18**) was a racemic naphthoquinone dimer, while rubiaquinones B (**19**) and C (**20**) were structurally unique trimeric naphthoquinones with a racemic nature possessing one chiral axis and one chiral carbon in common (Fig. 4) [20]. The absolute configurations of (+)-**18** and (–)-**18**, obtained by optical resolutions using chiral HPLC, were elucidated by interpretation of the ECD spectra with the aid of TDDFT ECD calculation. By contrast, the absolute configurations of optically active stereoisomers obtained from (±)-**19** and (±)-**20** were successfully assigned by analyses of the composite ECD spectra generated by summing ECD spectra of appropriate enantiomers. Racemic rubiaquinone A (**18**) exhibited antimicrobial activity against *Bacillus subtilis* (MIC 4 μg/mL).

**Fig. 5:**
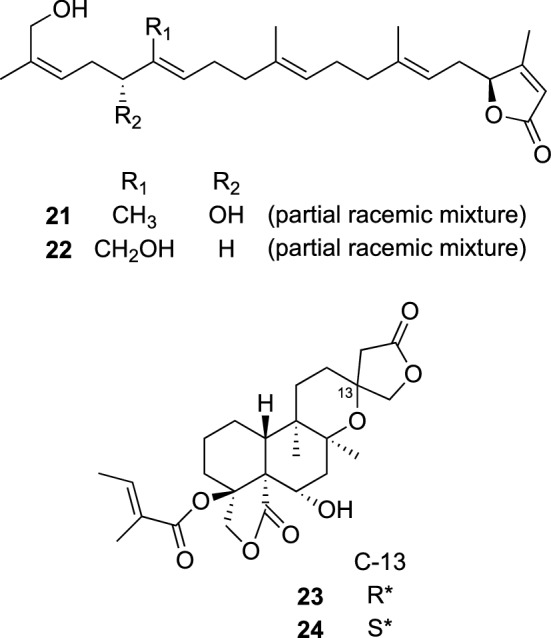
The structures of coleifolides A (**21**) and B (**22**) and scutefolides A1 (**23**) and A2 (**24**) isolated from *Scutellaria coleifolia*

*Scutellaria* plants, belonging to the Lamiaceae family, include about 350 species, and are widely distributed in temperate zone and tropical zone of Europe, North America, and East Asia. Many of them have been used as herbal medicines in various countries [21]. For example, the roots of *S. baicalensis* have been used as a pharmacopoeial medicine for removing fever and inflammation in Japan and China [22, 23], while the aerial parts of *S. galericulata* have been used as a sedative and an antispasmodic in United States [24]. Flavonoids, flavonoid glycosides and *neo*-clerodane type diterpenes were shown to be major constituents of the *Scutellaria* plants by previous studies. Biological activities including anticancer, anti-inflammatory and antifeedant activity of the isolated compounds were also reported [25]. We had an opportunity to learn that *S. coleifolia* is distributed in high altitude regions of Yunnan and Sichuan Provinces. The scientific studies of this plant have not been previously performed, although many biologically active compounds have been isolated from *Scutellaria* plants. Since finding new drug seeds from unexplored plant resources was also of our interest, we investigated the constituents of the aerial parts of *S. coleifolia*, which resulted in the isolation of two new sesterterpenes, coleifolides A (**21**) and B (**22**), and twenty-nine new diterpenes, together with six known compounds (Fig. 5). Coleifolides A (**21**) and B (**22**) were rare sesterterpenes from terrestrial plant [26], structurally similar to a marine sesterterpene, manoalides [27]. They were shown to be partial racemates, and the absolute configurations of their major isomers were assigned by analysis of the ECD spectra as well as NMR data for (*R*)- and (*S*)-methoxyphenylacetic ester derivatives. In contrast, twenty-nine new diterpenes include two acylated *neo*-clerodanes with a 19,18-γ-lactone, scutefolides A1 (**23**) and A2 (**24**) [28, 29]. Coleifolides A (**21**) and B (**22**) showed moderate cytotoxicity against four cancer cell lines, KB (epidermoid carcinoma), A549 (lung carcinoma), HeLa (uterine carcinoma), and MCF7 (breast carcinoma) cell lines, with IC_50_ value ranging from 13.6 to 37.6 μg/mL. In contrast, diterpenes exhibited weak or no cytotoxicity.

## Phytochemical studies on traditional herbal medicines used in Guangxi Zhuang Autonomous Region

Zhuang and Yao are two major minority groups in Guangxi Zhuang Autonomous Region, whose traditional medicines, Zhuang and Yao medicines, respectively, possessing over thousand years’ history, have been mainly used for the treatments of rheumatism, snakebite, malaria, cold, cancers, and skin problems. Approximately 4,000 medicinal plant species are seen in Guangxi, accounting for more than one-third of Chinese medicinal plant resources. Moreover, around 500 medicinal plants have been used as remedial agents in Zhuang medicine, while over 400 ones have been used in Yao medicine. These medicinal plants were selected mainly based on local flora, most of which were collected from the wild ecosystems [30]. We have been interested in these attractive traditional herbal medicines, and carried out phytochemical studies of them based on our collaborated ethnopharmacological study with researchers of the Guangxi Institute of Botany, Chinese of Academy of Sciences.

**Fig. 6:**
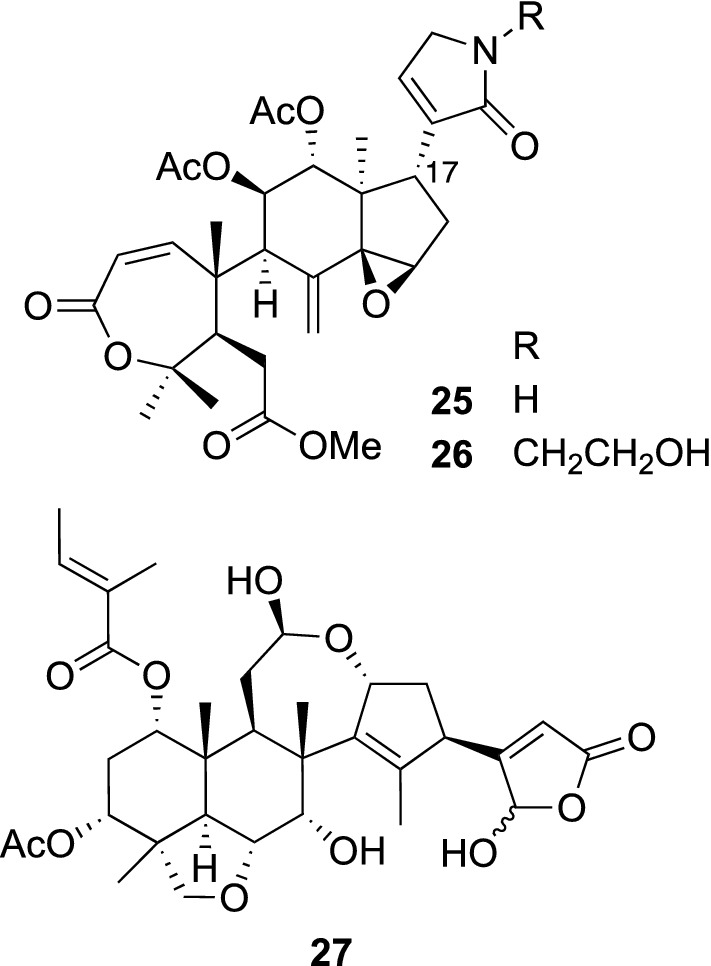
The structures of munropins A (**25**), B (**26**), and F (**27**) isolated from *Munronia pinnata*

Plants belonging to the genus *Munronia* (Meliaceae) are perennial herbs widely distributed in China, Sri Lanka, India, Indonesia, and the Philippines [31]. Among others, *M. pinnata* (Wall.) W. Theob. (synonyms: *M. henryi* Harms, *M. pumila* Wight, and *M. sinica* Diels) is a traditional herbal medicine used in China for the treatments of tuberculosis, cough, stomachache, and sores. Phytochemical investigation on the aerial parts of *Munronia pinnata* obtained at Jingxi, Guangxi gave six new limonoids including munropins A (**25**), B (**26**), and F (**27**) (Fig. 6) [32]. Munropins A (**25**) and B (**26**) possessed a prieurianin skeleton with α,β-unsaturated γ-lactam moieties at C-17. In contrast, munropin F (**27**) was assigned to have a nimbolinin type skeleton with a γ-hydroxy-α,β-unsaturated γ-lactone moiety. Their structures were assigned by detailed spectroscopic analyses.

**Fig. 7:**
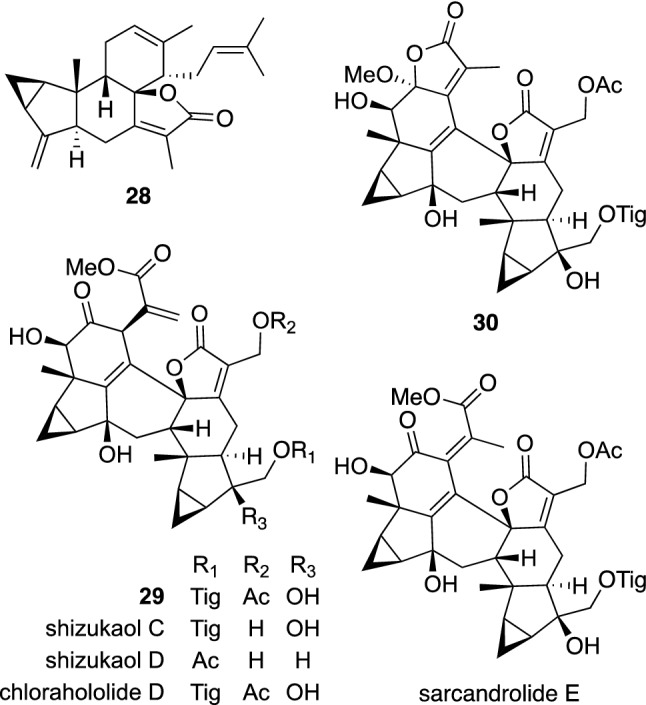
The structures of sarcaglabrins A–C (**28**–**30**), shizukaols C and D, chlorahololide D, and sarcandrolide E isolated from *Sarcandra glabra*

A Chloranthaceous plant, *Sarcandra glabra* (Thunb.) Nakai [synonym: *Chloranthus glaber* (Thunb.) Makino], is a perennial herb distributed in Japan, southern parts of mainland China, and southeastern Asia. The whole plants of *S. glabra* have been used as a traditional herbal medicine for the treatments of infectious disease and cancer in China [33]. The aerial parts of *S. glabra* collected Gongcheng, Guangxi were investigated to give new structurally interesting terpenoids, sarcaglabrins A–C (**28**–**30**) (Fig. 7) [34], together with 22 known related compounds including shizukaols C and D, chlorahololide D, and sarcandrolide E. Sarcaglabrin A (**28**) was assigned as a conjugate of lindenane type sesquiterpene and ocimene type monoterpene, while sarcaglabrins B (**29**) and C (**30**) were elucidated to be lindenane sesquiterpene dimers. In an evaluation of the antiproliferative activity against three human cancer cell lines, HeLa, MCF7, and A549, for the isolated compounds, shizukaols C and D, chlorahololide D, and sarcandrolide E were shown to exhibit weak cytotoxicities against Hela cells with IC_50_ values of 44.3, 43.0, 32.2, and 37.9 μM, respectively, while sarcandrolide E was also found to show a weak activity against MCF7 cells with an IC_50_ value of 46.5 μM. However, sarcaglabrins A–C (**28**–**30**) did not show cytotoxicity (IC_50_ > 50 μM).

## Phytochemical studies on other traditional herbal medicines used in China

**Fig. 8:**
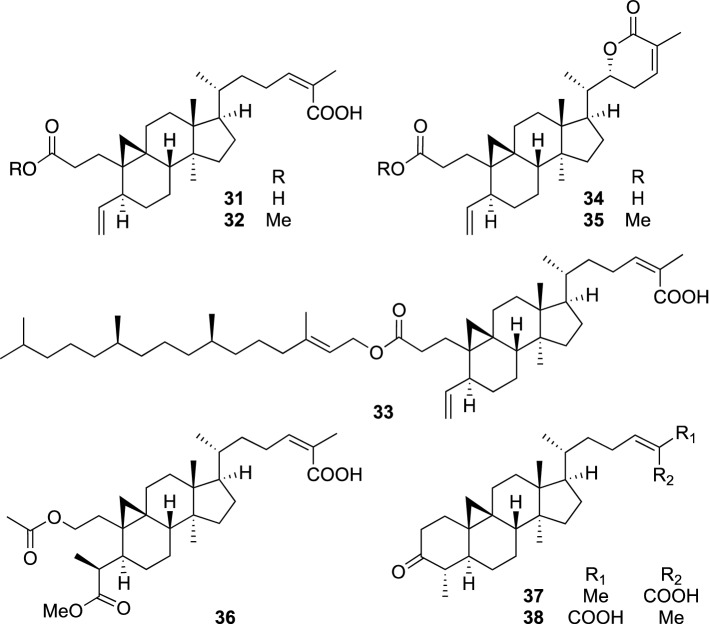
The structures of sinocalycanchinensins A–H (**31**–**38**) isolated from *Sinocalycanthus chinensis*

*Sinocalycanthus chinensis* Cheng and S.Y. Chang, the only representative in the genus *Sinocalycanthus* in the family Calycanthaceae, is native to Zhejiang of China. This plant wears attractive cream and yellow semi-double flowers on the terminal twigs from May to June, and therefore, is cultivated as an ornamental tree in China [35]. The leaves of *S. chinensis* are used as a remedy for cold, cough, and wheeziness [36], while the flowers and roots of this plant have been used for the treatment of stomachache [37]. We examined the leaves of *S. chinensis* and isolated eight new triterpenes, sinocalycanchinensins A–H (**31**–**38**) (Fig. 8) [38]. Sinocalycanchinensin F (**36**) represents the first example of a 29-*nor*-2,3-*seco*-cycloartane triterpene. In contrast, 29-*nor*-3,4-*seco*-cycloartane triterpenes were reported only from the aerial part of *Antirhea acutata* (Rubiaceae) in previous researches [39, 40], and sinocalycanchinensins A–E, G, and H appeared as the second examples of this type of triterpene. In addition, sinocalycanchinensin C (**33**) was the first example of a phytol ester of triterpene. Phytol, a component of chlorophylls, is known to be derived from a mevalonate-independent pathway, while cycloartanes are a metabolite of the mevalonate pathway. Sinocalycanchinensin C (**33**) is therefore a biosynthetically interesting molecule, as it is considered to be derived from the products of both mevalonate and mevalonate-independent pathways. Sinocalycanchinensins A–H (**31**–**38**) were evaluated for their cytotoxic activity against a panel of human cancer cell lines, KB, K562 (leukemia), and MCF7 cell lines, as well as multidrug-resistant (MDR) human cancer cell lines, including KB-C2 (colchicine-resistant KB) and K562/Adr (doxorubicin-resistant K562). Sinocalycanchinensin H (**38**) showed moderate cytotoxicities against all the tested cell lines, with IC_50_ values ranging from 14.8 to 18.8 μg/mL, while the cytotoxicities of sinocalycanchinensin G (**37**) were slightly less potent than those of **38**. Sinocalycanchinensin F (**36**) also showed similar cytotoxicities against the tested cell lines except for MCF7. In contrast, sinocalycanchinensin C (**33**) did not exhibit cytotoxicity against all the tested cell lines (IC_50_ values > 100 μg/mL). Sinocalycanchinensin E (**35**) was also a weak cytotoxic compound, but it showed significantly enhanced cytotoxicity against KB-C2 cells in the presence of colchicine with an IC_50_ value of 1.5 μg/mL. Since colchicine had no effect on the growth of KB-C2 cells at this concentration level, **35** might show some MDR-reversing effects. Overall, 3,4-*seco*-cycloartanes were less cytotoxic than the others.

**Fig. 9:**
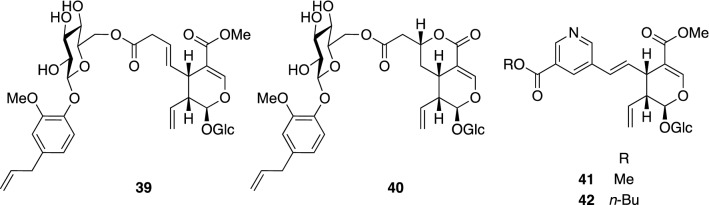
The structures of lonicerjaponins A (**39**) and B (**40**) and pyridinium alkaloid coupled secoiridoid glucosides (**41** and **42**) isolated from *Lonicera japonica*

*Lonicera japonica* Thunb. (Caprifoliaceae), also known as Japanese Honeysuckle, is a perennial twining woody vine native to the East Asia. This plant is often cultivated as an ornamental plant due to its attractive flowers and fragrances, while its flower buds have been used since early times for treatment of arthritis, diabetes mellitus, fever, infections, sores, and swelling [41]. The flower buds are also one of the major ingredients in Yin Qiao San, a most popular prescription in traditional Chinese medicine, used for common colds, including fever, headache, cough, thirst, and sore throat, and for influenza infection [42]. In an evaluation of the anti-influenza activity of Yin Qiao San in vitro, the H_2_O extract of Yin Qiao San inhibited all subtypes of influenza A, B, and C virus replications, and also exhibited inhibitory activity against an oseltamivir-resistant influenza A virus [43]. Therefore, chemical constituents of the flower buds of *L. japonica* were investigated, which resulted in the isolation of four new secoiridoid glucoside derivatives (**39**–**42**) (Fig. 9) [44], together with seven known secoiridoid glucosides including secoxyloganin and dimethylsecologanoside. Lonicerjaponins A (**39**) and B (**40**) are structurally unique conjugates of a secoiridoid glucoside with a phenolic glucoside, in which a 6"-*O*-malonyl moiety of the phenolic glucoside was considered to be involved in conjugation. In contrast, **41** and **42** were secoiridoid glucosides coupled with nicotinic acid derivatives. The inhibitory effect on the growth of the A/PR/8/34 influenza virus was evaluated by plaque assay. Secoxyloganin and dimethylsecolologanoside inhibited influenza A virus replication by ca. 50% at non-cytotoxic concentration (100 μg/mL), while a weak inhibitory activity was found in the other evaluated secoiridoid derivatives, except for **40**. However, none of the secoiridoid derivatives displayed influenza A neuraminidase (NA) inhibitory activity.

**Fig. 10:**
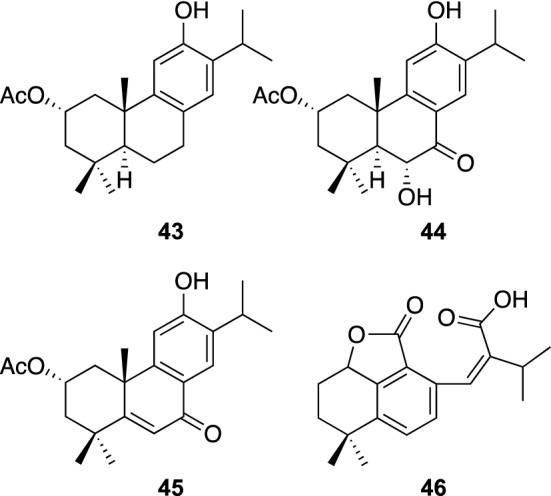
The structures of miltiorins A–D (**43**–**46**) isolated from *Radix Salviae miltiorrhizae*

Salviae miltiorrhizae Radix, the dried roots of *S. miltiorrhiza* Bge. (Lamiaceae) are one of the most popular traditional herbal medicines in Asian countries, and have been used extensively for the treatment of coronary artery disease, angina pectoris, myocardial infarction, cerebrovascular diseases, chronic renal failure, dysmenorrhea, and various types of hepatitis [45]. In our evaluation of the NA inhibitory effect of extracts from various herbal medicines, the MeOH extract from the dried roots of *S. miltiorrhiza* was found to exhibit an anti-NA activity. The MeOH extract was partitioned with CHCl_3_ and water, and the CHCl_3_-soluble material was found to show an NA inhibitory activity (IC_50_ 94.1 μg/mL). Repeated chromatographic separations of the CHCl_3_-soluble material afforded four new diterpenes, miltiorins A–D (**43**–**46**), together with eight known diterpenes including (+)-danshexinkun A (Fig. 10) [46]. Miltiorins A–C (**43**–**45**) are abietane diterpenes possessing a 2α-acetoxy group and a 12-hydroxy group in common, while **46** was a 11,12-*seco*-abietane diterpene with a γ-lactone ring. Miltiorin D (**46**) was the first example of the isolation of a 11,12-*seco*-abietane diterpene from natural sources. In an NA inhibitory assay for the isolated diterpenes, a norabietane diterpene, (+)-danshexinkun A, showed an NA inhibitory activity with an IC_50_ value of 39.5 μg/mL, while no other diterpenes exhibited NA inhibitory activities.

## Phytochemical studies on traditional herbal medicines used in Mongolia

Mongolia is located between China and Russia in the northern part of the Central Asian plateau, and has various climatic zones, including the northern taiga of Siberia, the steppe, and the deserts of Central Asia. Therefore, this country has a very diverse and distinctive flora, and many plants found there are unique. There are 3000 species of flowering plants, of which 975 species have been used traditionally in Mongolia and boundary countries [47]. The Mongolian medicine was influenced by Ayurveda and Tibetan medical systems. Mongolians have been using herbal remedies traditionally to prevent and cure the diseases of human and animals, and to improve animal productivity and fertility from ancient times [48]. About 72% of traditional Mongolian remedies were developed from plants, and over 28% were from animal or mineral sources [49], and various medicinal plants have been used in unique applications. We performed ethnobotanical and ethnopharmacological survey of Mongolian medicinal plants with researchers of Mongolian National University of Medical Sciences, Mongolia, based on the agreement between two universities.

**Fig. 11:**
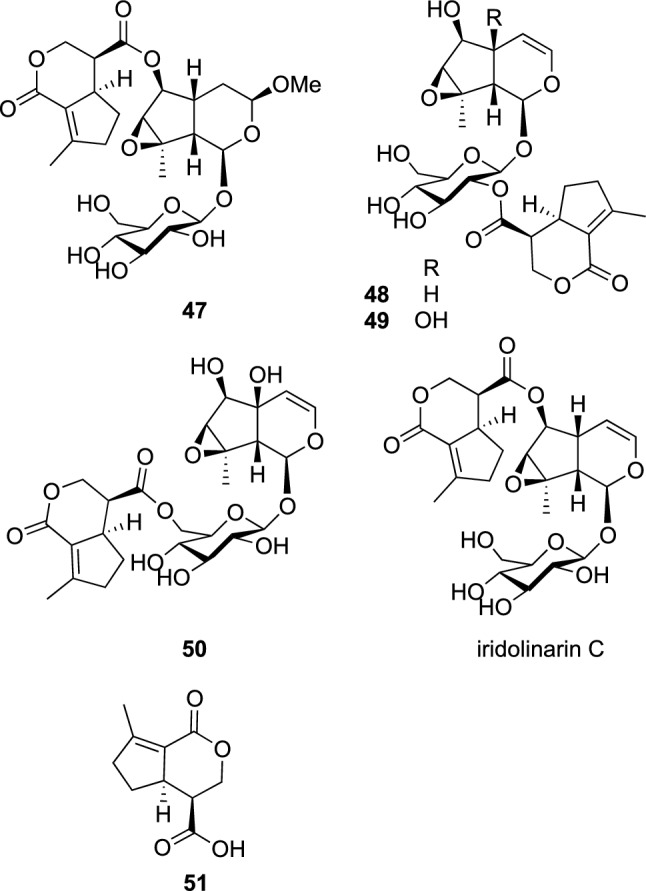
The structures of linaburiosides A‒D (**47**–**50**), 7-deoxyiridolactonic acid (**51**), and iridolinarin C isolated from *Linaria buriatica*

Plants of the genus *Linaria* (Plantaginaceae), composed of about 200 species, are mainly distributed in the Mediterranean Basin and Eastern Asia, and have been used medicinally as tonics, antiscorbutics, and antidiabetics [50]. *L. buriatica* Turcz. is a perennial herb distributed in central and eastern Siberia, and Mongolia [51]. In our ethnobotanical and ethnopharmacological survey in Mongolia, we learned that the flowers of *L. buriatica* have been used as an herbal medicine for the treatment of fever and edema. Phytochemical study on the aerial parts of *L. buriatica* resulted in the isolation of four hitherto undescribed acylated iridoid glucosides, linaburiosides A‒D (**47**–**50**), one undescribed iridoid, 7-deoxyiridolactonic acid (**51**) (Fig. 11) [52], and one known iridoid glucoside, iridolinarin C [53]. Linaburiosides A‒D (**47**–**50**), as well as iridolinarin C, had an acyl moiety corresponding to **51**. The absolute configuration of **51** was elucidated by application of the phenylglycine methyl ester (PGME) method, while those of **47**–**50** were assigned on the basis of chemical conversions, as well as application of the modified Mosher’s method. The absolute configuration of iridolinarin C was also elucidated in this study. Linaburioside A (**47**), iridolinarin C, and 7-deoxyiridolactonic acid (**51**), along with iridoid glucoside moieties of **49** and iridolinarin C (antirrinoside and 10-deoxycatalpol, respectively), 2′,3′:4′,6′-diacetonide derivative of iridolinarin C, and the metanolysate of **51** (7-deoxyiridolactonic acid dimethyl ester) showed an inhibitory effect on IL-1β production from LPS-stimulated microglial cells. Linaburioside A (**47**) and iridolinarin C significantly inhibited IL-1β production by 74.8 and 81.7%, respectively, at 100 μM with no cytotoxicity against microglial cells. In addition, the 2′,3′:4′,6′-diacetonide derivative of iridolinarin C suppressed IL-1β production by 91.3% at 100 μM more potent than **47** and iridolinarin C. From these results, acylated iridoid glucosides, **47** and iridolinarin C, appear to be a potential lead of therapeutic agent for neuroinflammation-related disease.

**Fig. 12:**
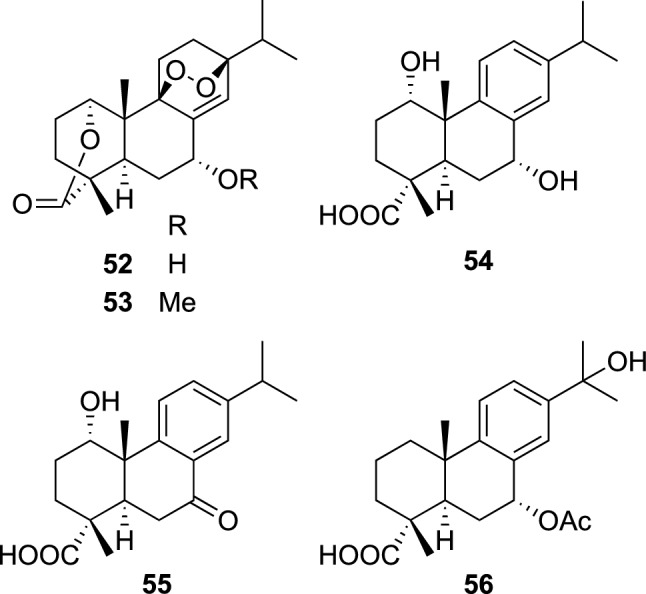
The structures of lophachinins A–E (**52**–**56**) isolated from *Lophanthus chinensis*

*Lophanthus* plants (Lamiaceae), composed of 23 species of perennial herbs or sub-shrubs, are distributed over alpine in Turkey, Iran, Afghanistan, central Asia, China, and Mongolia. Although the genus *Lophanthus* is considered to be very closely related to the well-studied genus *Nepeta* [54, 55], we learned in our survey that the aerial parts of *L. chinensis* Benth. have been used for the treatment of dizziness, fevers, and inflammatory diseases in Mongolia. Our chemical study on the aerial parts of *L*. *chinensis* afforded five new abietane diterpenes, lophachinins A–E (**52**–**56**), together with eleven known related diterpenes (Fig. 12) [56]. Based on a traditional usage of *L. chinensis* in Mongolia, all the isolated diterpenes were evaluated for their anti-inflammatory activity on microglial cells. Thus, lophachinins A (**52**) and B (**53**) demonstrated moderate inhibitory effects on IL-1β production at 100 μM by 81.6 and 48.2%, respectively, from LPS-treated microglial cells without cytotoxicity, while some known diterpenes also exhibited inhibitory effects comparable to **52** and **53**.

**Scheme 1:**
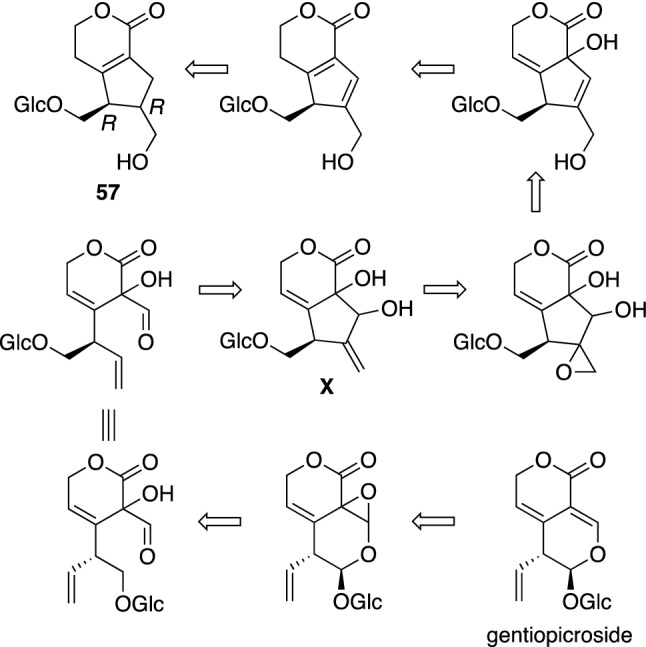
The structure of algiolide A (**57**) isolated from *Gentiana algida* and its plausible biogenetic pathway from gentiopicroside

*Gentiana algida* Pall. is a perennial plant distributed in North Asia and west area of North America. In Mongolia, the aerial parts of *G. algida* have been used for the treatment of throat illness caused by fever, lung disorders, liver disorders, and bile disorder [57]. Constituents of the aerial parts of *G. algida* were investigated to give a structurally unique secoiridoid glucoside, algiolide A (**57**). The structure of **57** was elucidated on the basis of spectroscopic analysis and chemical conversion as well as DFT calculation of ^13^C NMR and TDDFT calculation of ECD spectra [58]. Although a variety of secoiridoids were isolated from *Gentiana* plants, **57** has a novel skeleton consisting of fused α,β-unsaturated-δ-lactone and cyclopentene rings. Algiolide A (**57**) was considered to be derived from gentiopicroside, a major constituent of the flower of *G. algida* (Scheme 1). Thus, epoxidation of the double bond and cleavage of acetal, and intramolecular cyclization of gentiopicroside give a plausible intermediate (**X**), whose epoxidation followed by dehydration and reduction of the double bond yields algiolide A (**57**).

**Fig. 13:**
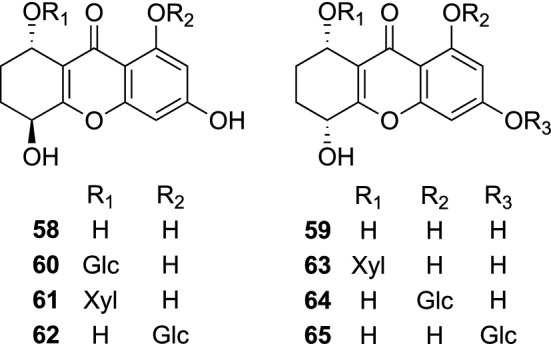
The structures of amarellins A–F (**60**–**65**) and their aglycones (**58** and **59**) isolated from *Gentianella amarella* ssp. *acuta*

Plants of the genus *Gentianella* (Gentianaceae) comprising about 250 species, of which many have a bitter taste, are distributed in temperate regions throughout the world [59]. *Gentianella amarella* ssp. *acuta* (synonym *Gentiana acuta*) is an annual herb distributed in East Asia, Siberia, and North America, and has been used as a traditional herbal medicine for the treatment of headache, fever, hepatitis, and gallbladder disorders in Mongolia [60]. The phytochemical investigation of the aerial parts of *G. amarella* ssp*. acuta* gave two tetrahydroxanthones, 1,3,5*S*,8*S*-tetrahydroxy-5,6,7,8-tetrahydroxanthone (**58**) and 1,3,5*R*,8*S*-tetrahydroxy-5,6,7,8-tetrahydroxanthone (**59**), and six new tetrahydroxanthone glycosides, amarellins A–F (**60**–**65**) [61]. Yang et al*.* also isolated **60**–**65** from the same plant materials, and evaluated for their inhibitory effects on the isolated intestinal smooth muscle contractions. All the evaluated compounds significantly reduced on contraction tension, while no significant change was observed for the isolated intestinal tissue contraction frequency [62] (Fig. 13).

**Fig. 14:**
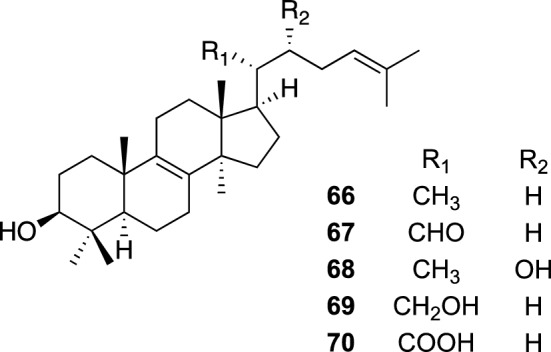
The structures of lanostane type triterpenes (**66**–**70**) as proproliferative agents on HFDPCs from the sclerotium of *Inonotus obliquus*

*Inonotus obliquus*, a fungus belonging to the Hymenochaetaceae family [63], is parasitic on birch trees found in Eastern and Northern Europe, Russia, Northern Asia, and in North America and Canada. The sclerotium of *I. obliquus*, also called Chaga mushrooms, has been used to treat cancer in Russia and Western Siberia for centuries. Although most scientific studies on Chaga mushrooms have been focused on their antitumor properties, we learned that the decoctions of Chaga mushrooms have been used traditionally in Mongolia as a shampoo for maintaining healthy hair. The information prompted us to study Chaga mushrooms for hair growth-promoting effects. Separation of the 80% EtOH aq. extract of Chaga mushrooms guided by a proproliferative activity on human follicle dermal papilla cells (HFDPCs) gave five lanostane-type triterpenes, lanosterol (**66**), lanost-8,24-diene-3β-ol-22-al (**67**), inotodiol (**68**), lanost-8,24-diene-3β,21-diol (**69**), and trametenolic acid (**70**). Proliferation rates of cells treated with **69** and **70** at 1.25 μM and with **69** at 5 μM were higher than those treated with 80 μM of minoxidil. Compounds **66** and **68** showed proproliferative effects at 0.31 and 20 μM, respectively, comparable to 80 μM of minoxidil, whereas **68** exhibited cytotoxicity at a higher concentration [64]. In contrast, **67** showed proproliferative effects at lower concentrations of 0.02 and 0.08 μM more potent than those treated with 80 μM of minoxidil, although proproliferative effects were not observed at concentrations over 0.31 μM. Thus, **66**–**70** appeared as potential candidates of new agents possibly used for hair care with a stimulative effect on hair growth. It should be noted that crude fractions obtained from Chaga mushroom extracts did not show inhibitory activities on testosterone 5α-reductase and androgen receptor, whereas several steroid derivatives with such activities are used for the treatment of alopecia [65]. This result suggested that these triterpenes have a mode of action related to hair growth different from those of steroid derivatives. The results of this study suggest that Chaga mushroom extracts could be utilized to an ingredient of a hair care product for the treatment of hair loss, and that triterpenes from Chaga mushrooms could be potential leads of therapeutic agents for promoting hair growth (Fig. 14).

## Phytochemical studies on traditional herbal medicines used in Uzbekistan

Uzbekistan, one of the republics in Central Asia, has rich source of plants. The flora of Uzbekistan includes more than 4500 species of vascular plants, of which 20% are endemic to Uzbekistan. Many of them are globally important but are endangered. The floristic data for several regions of Uzbekistan are still imperfect, and botanical studies are continuing. In addition, the phytochemical study and biological activity evaluation of these plants are still not fully conducted, and they may contain pharmaceutically potential constituents [66]. Characteristic herbal medicines only found in Uzbekistan have been used traditionally, since the number of endemic plants in this area is significantly higher than those of the other areas as described above. In addition, seven major ethnic populations with ancestors in Kazakhstan, Turkmenistan, Tajikistan, Afghanistan, Russia, Uzbekistan, and Kyrgyzstan, live in Uzbekistan, and therefore, the wide spectrum of medicinal plant utilizations are found based on ethnic differences [67]. About 70% of Uzbek households use traditional herbal medicines in their daily lives [68]. Our ethnopharmacological survey of Uzbek traditional herbal medicines and their phytochemical studies have been performing based on ethnopharmacological information exchanges with the researchers at Institute of Botany and Botanical Garden, Academy of Science of Uzbekistan and Institute of the Chemistry of Plant Substances, Academy Sciences of the Republic of Uzbekistan.

**Fig. 15:**
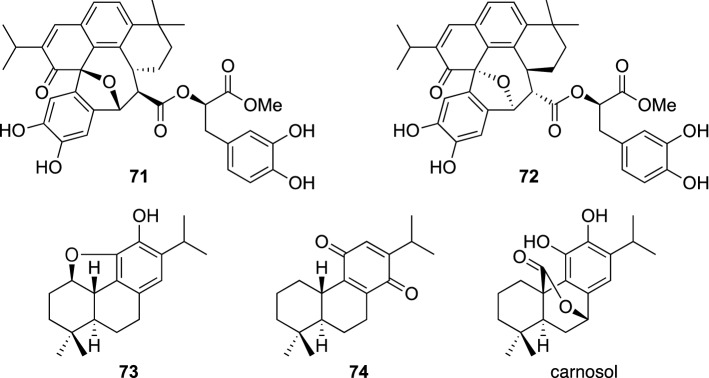
The structures of perovsfolins A–D (**71**–**74**) and carnosol isolated from *Perovskia scrophulariifolia*

About 100 Lamiaceous plants are considered to be native of Uzbekistan, while plants of the genus *Perovskia*, a small group of the Lamiaceae family, are aromatic shrubs growing in arid region of Central Asia [69]. The aerial parts of *P. scrophulariifolia* have been used as a traditional herbal medicine to treat dermatitis and human intestinal parasites in Uzbekistan. The aerial parts of *P. scrophulariifolia* have been investigated, which resulted in the isolation of two novel terpenoids, perovsfolins A (**71**) and B (**72**) [70], and two new 20-norabietane diterpenes, perovsfolins C (**73**) and D (**74**) [71] (Fig. 15), together with 13 known diterpenes including 7-*O*-methylrosmanol, carnosol, and demethylsalvicanol quinone, as well as methylrosmarinate. Perovsfolins A (**71**) and B (**72**) possessed a C_28_ terpenoid moiety with unprecedented 6/8/6/6/6 pentacyclic carbon skeleton, which were presumed to be generated by condensation of a 20-norabietane diterpene and methyl rosmarinate, where spontaneous (non-enzymatic) reactions may be involved. Perovsfolins A–D (**71**–**74**) and known diterpenes were evaluated for their inhibitory activity on IL-1β production from LPS-stimulated microglial cells as part of our search for natural products to be a potential lead of therapeutic agent for neuroinflammation-related disease. Thus, carnosol potently inhibited IL-1β production by 96% from LPS-stimulated microglial cells at 12.5 μM without cytotoxicity (cell viability > 80%) against microglial cells. Columbaridione, 7-*O*-methylrosmanol, and demethylsalvicanol quinone also showed inhibitory activities (45.9% at 6.25 μM, 41.5% at 12.5 μM, and 44.8% at 25 μM, respectively) with no cytotoxicity. Perovsfolin B (**72**) was also found to be a weak inhibitor of IL-1β production from LPS-stimulated microglial cells (24.3% at 25 μM) without cytotoxicity against microglial cells (cell viability > 80%).

**Fig. 16:**
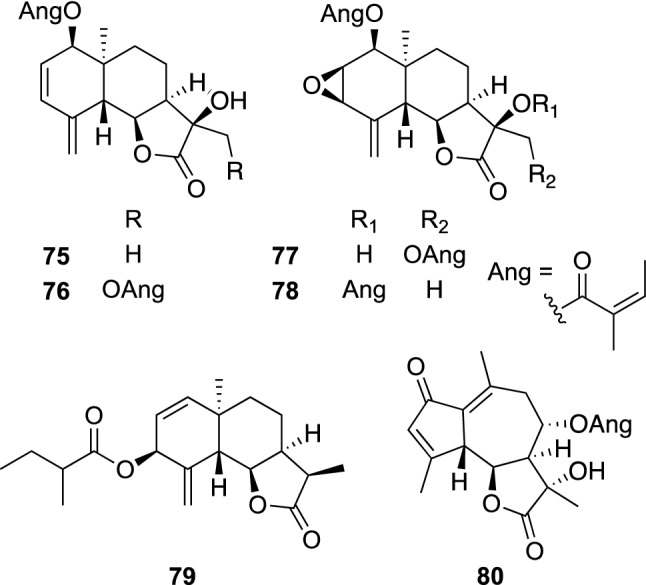
The structures of acylated sesquiterpenes (**75**–**80**) isolated from *Ferula varia*

The genus *Ferula*, one of the largest genus of the Apiaceae (Umbelliferae) family, has about 170 species distributed from Central Asia, throughout the Mediterranean region to northern Africa. Several species of this genus have been used in ethno-medicine for the treatment of digestive disorders, headache, dizziness, arthritis, rheumatism, and toothache and as tranquilizers in Uzbekistan. The roots of *F. varia* (Shrenk) Trautv. have been used traditionally to treat fever and intestinal parasites and as a mouth rinse. The MeOH extract from the roots of *F.* *varia* was partitioned with EtOAc and H_2_O. Repeated chromatographic separations of the EtOAc-soluble materials gave six new (**75**–**80**) and five known sesquiterpene lactones [72], while the H_2_O-soluble materials was chromatographed to give fifteen new sesquiterpene lactone glycosides, including twelve new eudesmane, two new guaiane, and one new germacrane type sesquiterpene lactone glucosides [73, 74]. All the isolated compounds had a *cis*-fused γ-lactone ring at C-6 and C-7, which was also seen in sesquiterpene lactones isolated from the EtOAc-soluble fraction. *F. varia* characteristically contained sesquiterpene lactone derivatives with a *cis*-fused γ-lactone. The sesquiterpenes isolated from the EtOAc-soluble fraction of *F. varia* were evaluated for their cytotoxic activity against human tumor cell lines, including MDR human cancer cell lines KB-C2 and K562/Adr. Compound **78** was significantly cytotoxicity against KB-C2 cells with an IC_50_ value of 15.7 μg/mL, while doxorubicin, tested as a positive control, was nontoxic against the resistant cell lines (> 100 μg/mL). Cytotoxicity of **78** against KB-C2 was selective, since it was 4.6 times more potent than that (IC_50_ 72.8 μg/mL) against sensitive cells (KB). Compound **78** was also slightly more cytotoxic against K562/Adr cells than against K562 cells. Compounds **75**, **77**, and **79**, and 8α-angeloyloxy-10β-hydroxy-3-en-6,12-olide were nontoxic against all the tested cell lines (IC_50_ values were > 100 μg/mL), whereas they showed moderate cytotoxicity against KB-C2 cells with IC_50_ values ranging from 25.4 to 67.8 μg/mL in the presence of 2.5 μM colchicine, indicating that these compounds have some MDR-reversing effect. Since MDR inhibitors are known to inhibit the efflux of anticancer drugs by interfering with P-glycoprotein (P-gp) function, compounds **77** and **78**, and 8α-angeloyloxy-10β-hydroxy-3-en-6,12-olide were also examined for their effects on P-gp function in KB-C2 cells. Compounds **77** and **78**, and 8α-angeloyloxy-10β-hydroxy-3-en-6,12-olide increased rhodamine 123 accumulation to 169 and 134, and 114%, respectively, at 50 μM, while verapamil, a modulator of P-gp function, showed an increase of rhodamine 123 accumulation by 32% at 2 μM and by 80% at 5 μM. Since these sesquiterpenes had no effect on the efflux of rhodamine 123 from KB-C2 cells by P-gp, **77** and **78** were considered to change the toxicity of colchicine against KB-C2 cells by other than interfering with P-gp function (Fig. 16).

**Fig. 17:**
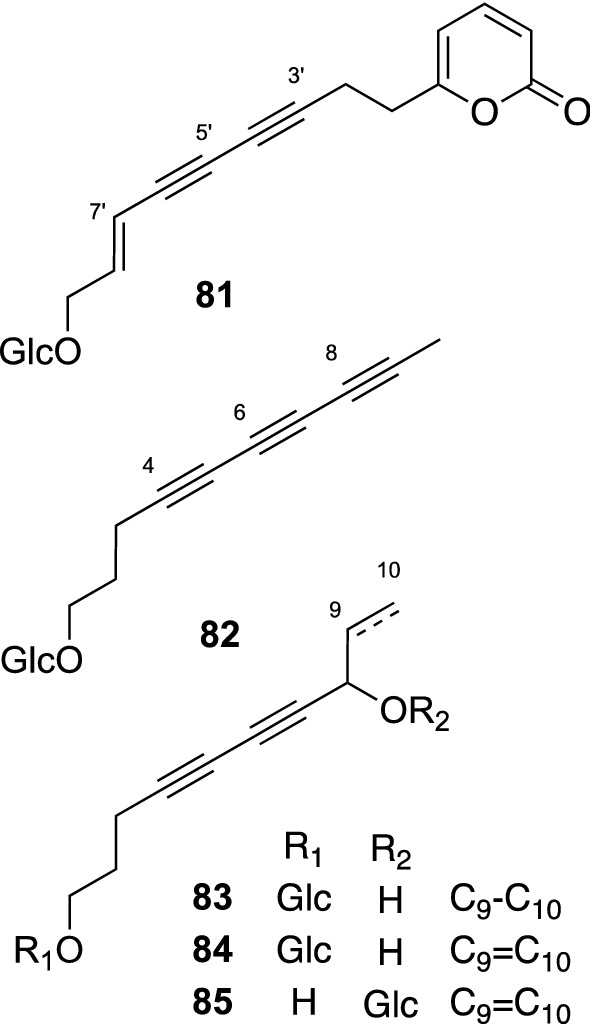
The structures of polyacetylenic glucosides (**81**–**85**) isolated from *Mediasia macrophylla*

*Mediasia macrophylla* (Regel ex Schmalh.) Pimenov (Apiaceae) is an Uzbek medicinal plant, whose aerial parts have been used traditionally as a perfume, an appetite enhancer, a natural preservative, and for treatment of rheumatism, nephritis, eczema, herpes, and injury [75]. This plant is also used in combination with four other medicinal plants, *Ziziphora pedicellate* (Lamiaceae), *Urtica dioica* (Urticaceae), *Codonopsis clematidea* (Capmpanulaceae), and *Origanum tyttanthum* (Lamiaceae), for the improvement of hepatic function in Uzbekistan. Our phytochemical study on the aerial parts of *M. macrophylla* furnished five new polyacetylenic glucosides (**81**–**85**) (Fig. 17) [76]. Polyacetylenic compounds are widely distributed especially in the families Apiaceae, Asteraceae, and Araliaceae, and fungi of the group Basidiomycetes. Among these, Apiaceous plants mainly contain C_17_-polyacetylenic compounds [77]. By contrast, C_10_- and C_14_-polyacetylenic glycosides have been reported only from a few members of the Asteraceae and Campanulaceae families [78]. Compounds **82**–**85** appear to be the first example of C_10_-polyacetylenic glucosides from an Apiaceous plant. In addition, compound **81** was the first example of a polyacetylen derivative possessing an α-pyrone moiety from natural sources. Later, Jelodarian et al. reported the isolation of polyacetylene containing an α-pyrone moiety from *Echinophora cinerea* in 2017 [79]. The co-occurrence of C_10_-polyacetylenic glucosides (**82**–**85**) and a C_14_-polyacetylenic glucoside possessing an α-pyrone moiety (**81**) suggested that the C_14_-polyacetylenic unit of **81** may be derived from a C_10_-polyacetylenic compound, such as the aglycone of **82**, by condensation of the additional two malonyl units, followed by oxidation. Compound **84** as well as its aglycone were later isolated from *Artemisia capillaris* (Apiaceae) by Geng et al*.*, and the 8*S*-configuration of aglycone was deduced from its specific rotation. In addition, an inhibitory activity of **84** against HBV DNA replication with an IC_50_ value of 11.0 μg/mL (SI = 79.3) was also reported [80].

**Fig. 18:**
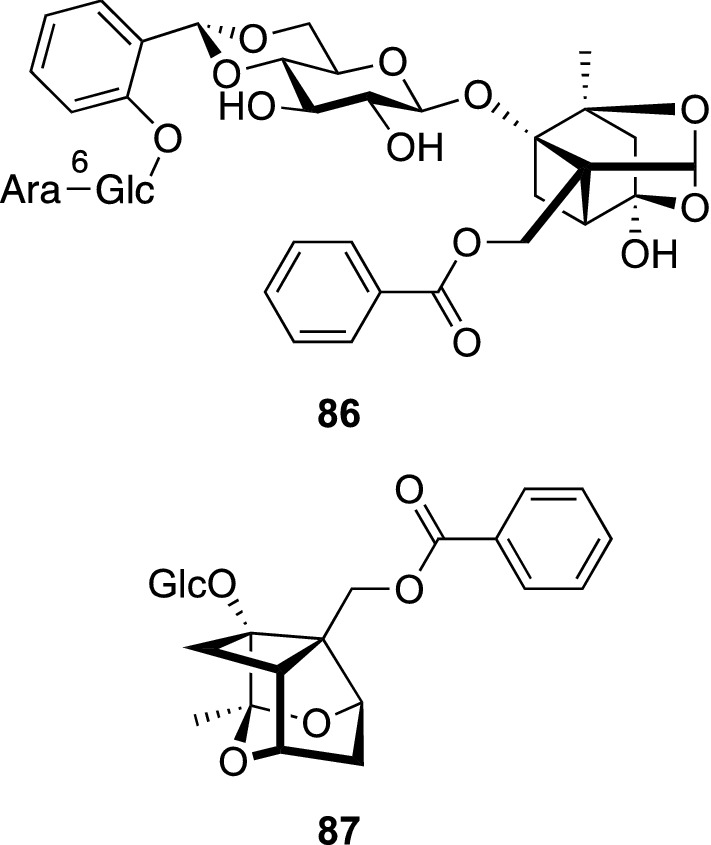
The structures of paeonihybridin (**86**) and paeobrin (**87**) isolated from *Paeonia hybrida*

**Scheme 2:**
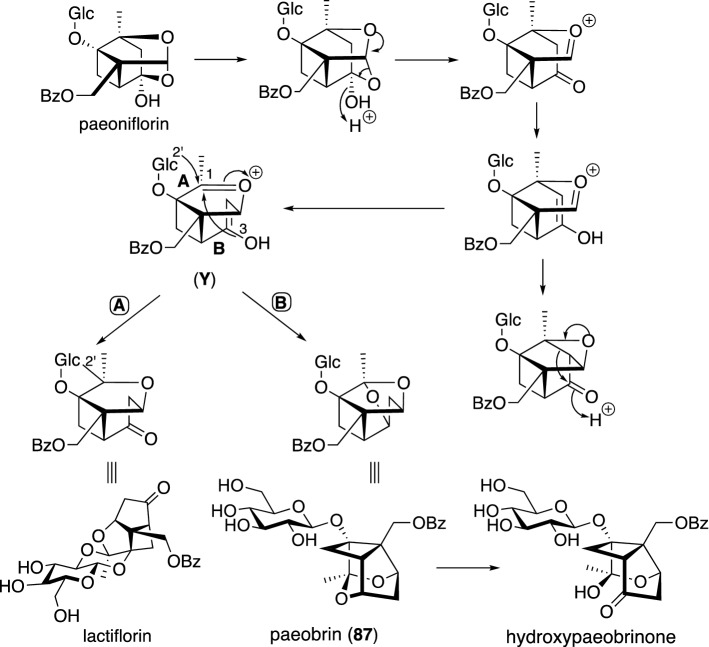
Possible biosynthetic pathway of paeobrin (**87**), hydroxypaeobrinone, and lactiflorin from paeoniflorin

Paeoniae Radix, one of the most important traditional herbal medicines, has been used for remedies for gynecological problems, cramps, pain, and dizziness in Japan and China. A variety of paeoniflorin-related monoterpene glycosides and tannins were shown to be characteristic metabolites of *Paeonia* plants (Paeoniaceae) [81]. *P. hybrida* Pall. is a traditional herbal medicine used in Uzbekistan, and a water decoction of its roots has been used to treat nerve disease [82]. We examined phytochemically on the roots of *P. hybrida* to isolate six new monoterpene glycosides including paeonihybridin (**86**) and paeobrin (**87**) as well as fourteen known compounds (Fig. 18) [83]. Paeonihybridin (**86**) had a unique hybrid structure consisting of paeoniflorin and paeonovicinoside. Paeobrin (**87**), possessing a novel carbon framework, was considered to be derived from paeoniflorin through the similar process for the production of lactiflorin [84] as shown in Scheme 2. Thus, an oxacyclic ring-opening of paeoniflorin followed by successive cationic 1,2-carbon shifts yield a plausible biogenetic intermediate (**Y**), which produces lactiflorin or paeobrin depending on whether C-1 forms an ether bond with C-2′ in the glucosyl moiety or C-3, respectively. Later, Ha et al. reported the isolation of hydroxypaeobrinone, whose structure is closely related to **87**, from *P. suffruticosa* [85].

**Fig. 19:**
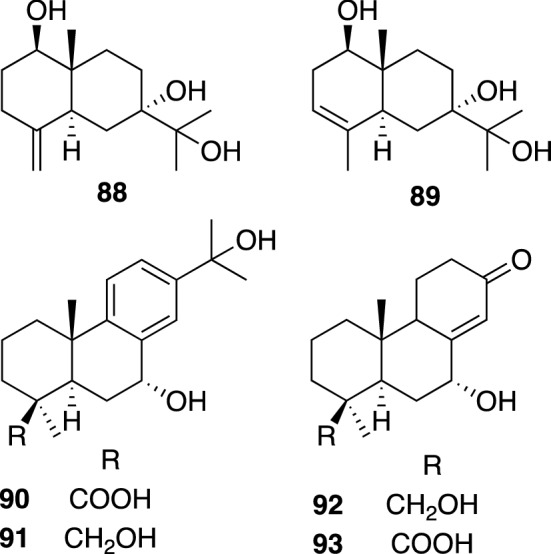
The structures of new sesquiterpenes (**88** and **89**) and diterpenes (**90**–**93**) isolated from *Juniperus polycarpus* var. *seravschanica*

*Juniperus* plants (Cupressaceae), including about 70 species, are evergreen shrubs or trees, and are widely distributed over the northern hemisphere. Some *Juniperus* species have been traditionally used in medicinal purposes; *J. communis* L. has been used for the treatment of tuberculosis, while *J. oxycedrus* L. has been used in Turkey as an ethnic herbal medicine for the treatment of diabetes [86]. *J. polycarpus* var. *seravschanica* (syn. *J. seravschanica*) is an evergreen tree indigenous to the mountains of Central Asia. Its seed decoction has been used as an herbal medicine for kidney diseases, and as a diuretic and abortive in Uzbekistan [87, 88]. Repeated chromatographic separations of the extract of the air-dried fruits of *J. polycarpus* var. *seravschanica* afforded two new sesquiterpenes (**88** and **89**), and four new diterpenes (**90**–**93**), together with nine known compounds including cedrol and sugiol (Fig. 19) [89]. In evaluation of an antimalarial activity against three *Plasmodium falciparum* clones, D6, TM91C235, and W2, cedrol and sugiol demonstrated weak activities against D6 clone (IC_50_ values of 0.059 and 0.47 μg/mL, respectively), TM91C235 clone (0.30 and 1.0 μg/mL), TM91C235 clone (0.30 and 1.0 μg/mL), and against W2 clone (0.22 and 0.41 μg/mL), while the other tested compounds did not show significant antimalarial activity.

**Fig. 20:**
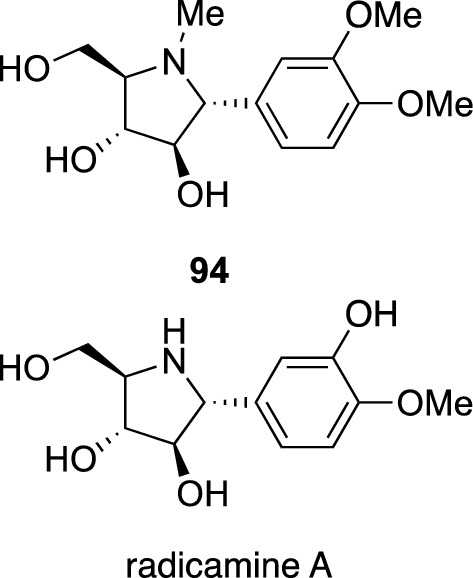
The structures of codonopsinol (**94**) isolated from *Codonopsis clematidea* and radicamine A

Plants of the genus *Codonopsis* (Campanulaceae), commonly called as bonnet bellflower, are perennial herbs endemic to East Asia. The roots of several *Codonopsis* species have been used in Chinese traditional medicine as *Codonopsis* Radix to treat appetite loss, diarrhea, and vomiting. In contrast, the aerial parts of *C. clematidea* (Schrenk) Clark have been used in Uzbekistan to treat liver disease, hepatitis, and jaundice. This plant is also used in combination with four other medicinal plants to improve hepatic function as described above. Examination of the aerial parts of *C. clematidea* resulted in the isolation of a new codonopsine-related alkaloid, codonopsinol (**94**), together with thirteen known compounds [90]. Codonopsinol (**94**) being structurally related to radicamine A, an α-glucosidase inhibitor isolated from *Lobelia chinensis* (Campanulaceae) [91], was expected to have similar activity. Synthetic study of a series of 2-aryl polyhydroxylated pyrrolidines, including (–)-**94** and (–)-radicamine A, as well as their evaluation of inhibitory activity against α-glucosidase was carried out by Cheng et al. (–)-Codonopsinol (**94**) was shown to have an inhibitory activity against α-glucosidase of yeast and *Bacillus stearothermophilus* lyoph, although its potency was about half of radicamine A [92] (Fig. 20).

## Phytochemical studies on traditional herbal medicines used in Bangladesh

Bangladesh, a subtropical country of Indian sub-continent, possess a great diversity in plants, being estimated to have about 7000 endemic plant species, including bryophytes, pteridophytes, gymnosperms, and angiosperms [93]. There are approximately 5,000 angiosperms, belonging to about 200 families, distributed of which 500 have been used in the traditional medical systems including Ayurvedic and Unani for the treatment of different types of diseases [94]. In addition, Bangladesh has the Board of Unani and Ayurvedic Systems of Medicine, which arrange for the standardization of Unani and Ayurvedic systems of medicine, and these established are widely used in this country [95]. Besides these medical systems, traditional medicinal practitioners, known variously as Kavirajes or Vaidyas, in Bangladesh have been used their own herbal medicines [96]. We have been studying traditional herbal medicines for searching new drug leads based on ethnopharmacological information exchanges with the researchers of Jahangirnagar University.

**Fig. 21:**
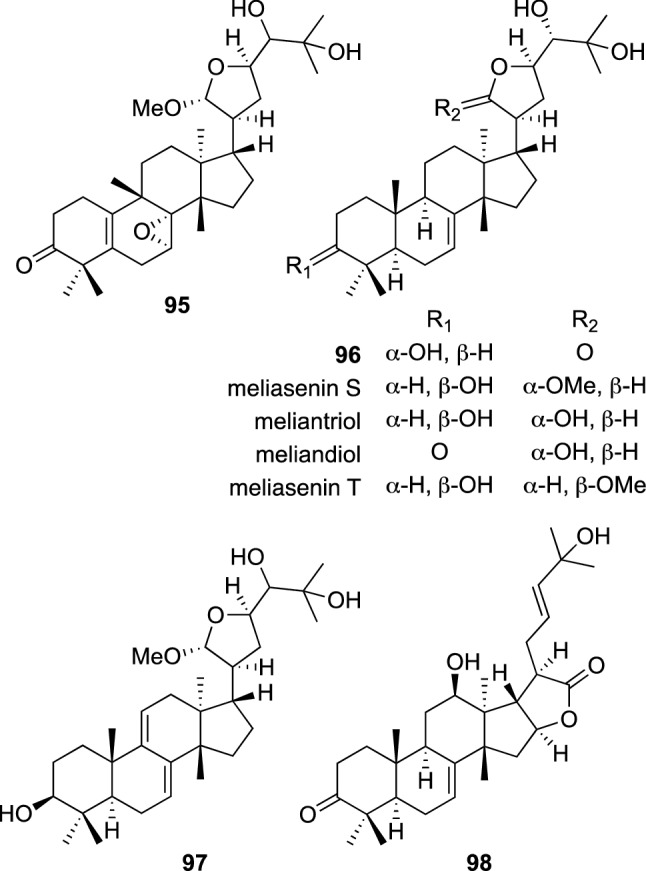
The structures of indicalilacols A–D (**95**–**98**), meliasenin S, meliantriol, and melianodiol isolated from *Azadirachta indica*, and meliasenin T

*Azadirachta indica* A. Juss (Meliaceae), also known well as neem tree, is widely distributed in the tropical zones of Africa, South Asia, and India. This plant has been used as a traditional herbal medicine in India for more than 2000 years, because of its valuable biological activities, especially its anti-inflammatory, anti-ulcer, antimalarial, antibacterial and antioxidant activities. *A. indica* is also known to contain an antifeedant limonoid, azadirachtin A [97, 98]. Our phytochemical study on the fruits of *A. indica* furnished four new molecules, indicalilacols A–D (**95**–**96**) (Fig. 21), which were assigned as a 19(10 → 9β)*abeo*-tirucallane triterpene (**95**), tirucallane triterpenes (**96** and **97**), and a euphane triterpene (**98**), as well as three known tirucallane type triterpenoids, meliasenin S, meliantriol, and melianodiol [99]. Cytotoxicity against two human tumor cell lines (KB and MCF7) and against one MDR cancer cell line (KB-C2) was evaluated for the isolated compounds, together with meliasenin T, prepared from meliantriol. Indicalilacol (**96**), meliantriol, melianodiol, and meliasenin T were moderately cytotoxic against all the tested cell lines with IC_50_ values ranging from 9.04 to 25.7 μg/mL, while meliasenin S showed no cytotoxicity against any of the tested cancer cell lines. However, meliasenin S might have an MDR-reversal effect, since it showed cytotoxicity against KB-C2 cells in the presence of 2.5 μM colchicine with an IC_50_ value of 6.48 μg/mL. In contrast, meliasenin T, the C-21 epimer of meliasenin S, only showed a week cytotoxicity against KB-C2 cells in the presence of 2.5 μM colchicine, and therefore, the stereochemistry of C-21 might play an important role.

**Fig. 22:**
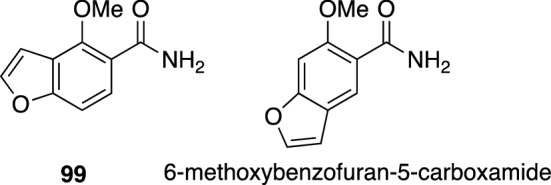
The structures of benzofuran derivative (**99**) isolated from *Tephrosia purpurea* and its isomer as anti-allergic agents

*Tephrosia purpurea* (L.) Pers. (Fabaceae), also commonly known as “sarpunkha” and “wild indigo”, is a perennial herb widely grown in India, Ceylon, Mauritius, tropical Africa and subtropical regions. Various parts of this plant have been used as tonic, laxative, and diuretic as well as for the treatment of bronchitis, bilious febrile attack, boils, pimples, diarrhea, gonorrhea, rheumatism, and disease of heart, spleen, and blood in Ayurvedic medical system [100]. In addition, *T. purpurea* has been used in the Unani medical system for the treatment of skin and other disorders associated with morbid blood, and also used as an important ingredient in blood purifier formulations [101]. In the course of our search for anti-allergic natural products from traditional herbal medicines [102, 103], *T. purpurea* was investigated based on bioassay-guided fractionation and isolation, which resulted in the isolation of an active principle. The structure of this compound was elucidated by spectroscopic analysis and chemical synthesis to be 4-methoxybenzofuran-5-carboxamide (**99**) (Fig. 22) [104]. An isomer of **99**, 6-methoxybenzofuran-5-carboxamide, together with their demethyl derivatives, as well as derivatives whose carboxamide replaced with carboxylic acid, were also synthesized, and their activity on phorbol 12-myristate 13-acetate (PMA)-induced H1R gene expression in HeLa cells was evaluated. Compound **99** suppressed H1R mRNA up-regulation in HeLa cells with an IC_50_ value of 75.3 μM, while its isomer, 6-methoxybenzofuran-5-carboxamide, suppressed H1R gene expression with an IC_50_ value of 49.1 μM. Among the evaluated derivatives, only 6-methoxybenzofuran-5-carboxylic acid showed similar level of activity (IC_50_ 84.6 μM). Mechanism study of this compound revealed that **99** ameliorated allergic symptoms and suppressed the elevation of H1R and helper T cell type 2 (Th2) cytokine mRNAs in TDI-sensitized rats. Further study suggested that the mechanism of **99** for H1R gene suppression underlies the inhibition of PKCδ activation. In addition, it was shown that **99** alleviates nasal symptoms in TDI-sensitized rats through the inhibition of H1R and Th2 cytokine gene expression [105].

**Fig. 23:**
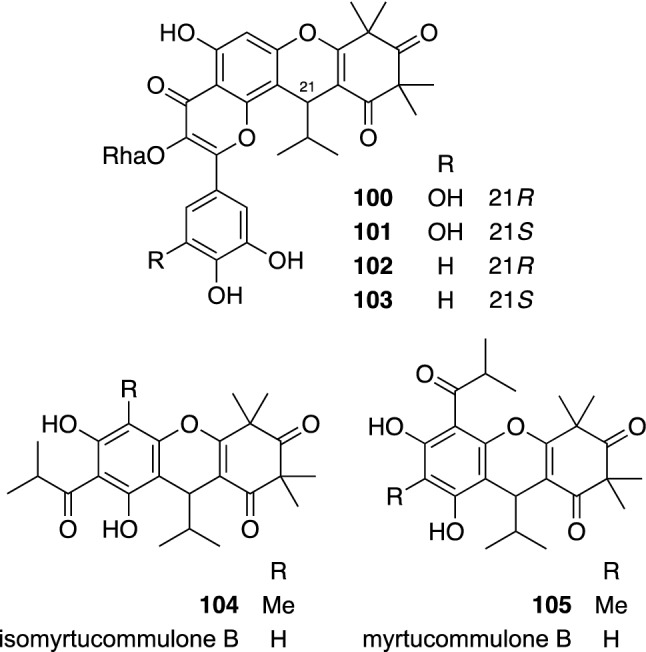
The structures of myrtucommunins A–D (**100**–**103**), phloroglucinol dimers (**104** and **105**), and isomyrtucommulone B, and myrtucommulone B isolated from *Myrtus communis*

*Myrtus communis*, an evergreen sclerophyll shrub or small tree belonging to the Myrtaceae family, is widely distributed from the northwestern to the eastern Mediterranean, including bordering countries and western Asia [106]. This plant has been used as a spice, as well as for medicinal and food preparation purpose in various parts of the world since ancient Greece period, and is one of the important drugs being used in Unani and Ayurveda systems [107, 108]. Chemical constituents of the leaves of *M. communis* were investigated to give seven new phloroglucinol derivatives, including myrtucommunins A–D (**100**–**103**) and 6-methylisomyrtucommulone B (**104**) and 4-methylmyrtucommulone B (**105**) (Fig. 23), together with six known compounds including isomyrtucommulone B and myrtucommulone B. Myrtucommunins A–D (**100**–**103**) were assigned as conjugates of polymethylated acylphloroglucinol and flavonol L-rhamnoside, while 6-methylisomyrtucommulone B (**104**) and 4-methylmyrtucommulone B (**105**) were assigned as 6/6/6 tricyclic acylphloroglucinol derivatives with a racemic nature [109]. Racemic isomyrtucommulone B exhibited significant antimicrobial activities against *Staphylococcus aureus* including methicillin-resistant strains and *Bacillus subtilis*. Comparable antimicrobial activities were also found in the enantiomers (+)-isomyrtucommulone B and (–)-isomyrtucommulone B obtained by the chiral separation, suggesting that the stereochemistry is not essential for its antimicrobial activity.

**Fig. 24:**
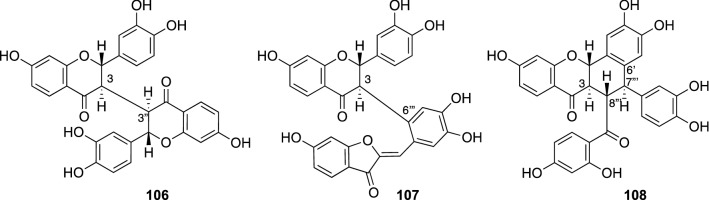
The structures of new biflavonoids (**106**–**108**) from isolated *Butea monosperma*

*Butea monosperma* (Lam.) Taub. (Fabaceae), commonly known as ‘Flame of forest’ or ‘Palash’, is a small to medium-sized deciduous tree widely distributed in tropical southern Asia up to an altitude of 4000 ft. Its roots have been used for elephantiasis and several eyesight defects, while the bark decoction has been used for the treatment of goiter, ulcer, dysentery, liver disorder and tumors [110]. In contrast, its flowers have been used as an emmenagogue, diuretic, depurative, and tonic, as well as for the treatment of leprosy, gout and skin diseases, spermatorrhea, and leucorrhoea [111]. The flowers of this plant are also used commercially as a natural dyeing due to their bright orange-red color. Several biological activities for the flowers of *B. monosperma* have been reported including antidiabetic, anticonvulsive, antiesterogenic, antioxidative, hepatoprotective, chemopreventive and anti-inflammatory activities, and some of which validated the traditional use of this plants [112]. However, their active components have not been clarified. We examined the flowers of *Butea monosperma*, resulting in the isolation of a new aurone glucoside and three new biflavonoids and some known flavonoids including butein, sulfurein, and monospermoside, coreopsin. The new aurone glucoside was characterized to be sulfuretin 3-*O*-β-glucopyranoside, while new biflavonoids were assigned as butin 3,3″-dimer (**106**), an auronoflavanone type biflavonoid (**107**) and a chalconoflavanone type biflavonoid (**108**), respectively. Compound **107** was the only auronoflavanone type biflavonoid linked between C-3 and C-6′, while **108** was the first chalconoflavanone type biflavonoid linked between C-3 and C-8″″, and between C-2′ and C-7″″ (Fig. 24) [113]. In an influenza A neuraminidase inhibitory assay, butein displayed relatively potent inhibitory activity with an IC_50_ value of 5.4 μg/mL. Sulfuretin 3-*O*-β-glucopyranoside, monospermoside, and coreopsin showed moderate influenza A neuraminidase inhibitory activity with IC_50_ values ranging from 28.5 to 34.9 μg/mL. By contrast, compound **108** showed potent activity in a DPPH free-radical scavenging assay with an IC_50_ value of 1.7 μg/mL.

**Fig. 25:**
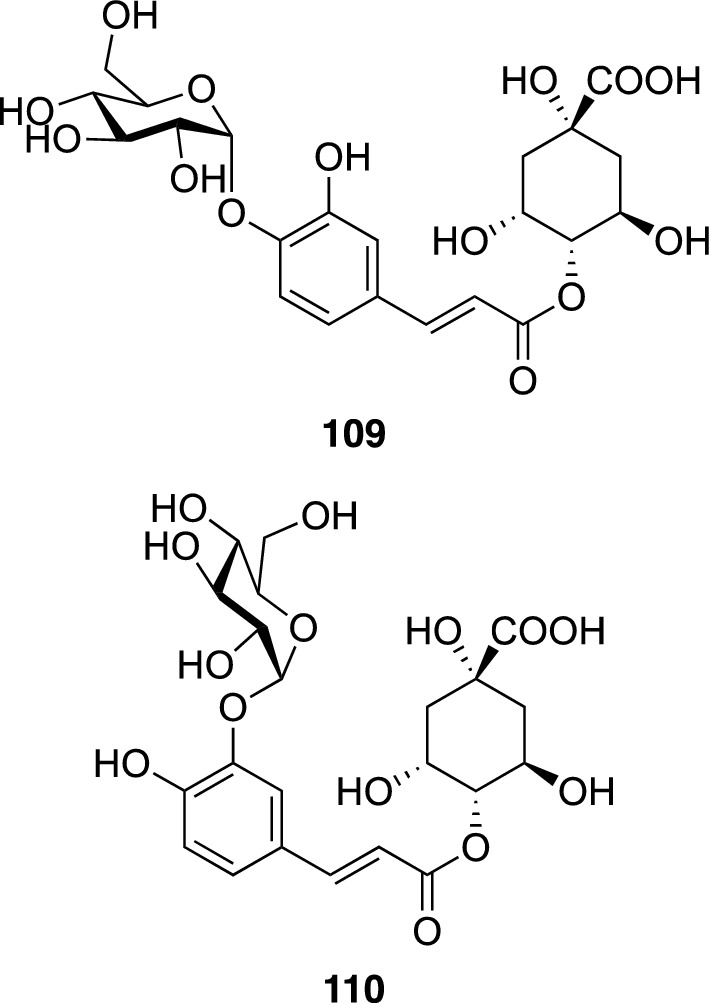
The structures of α-D-glucopyranosyl caffeoyl quinic acids (**109** and **110**) isolated from *Moringa oleifera*

An Ayurvedic medicinal plant, *Moringa oleifera* Lam. (Moringaceae), is widely distributed in tropical areas [114]. The roots of *M. oleifera* is listed in the Indian Materia Medica as useful for the treatment of a number of ailments, including asthma, gout, lumbago, rheumatism, enlarged spleen or liver, internal deep-seated inflammations and calculi [115, 116]. In contrast, the leaves, fruit, flowers and immature pods of this plant are used as a nutritive vegetable in India, Bangladesh, Pakistan, the Philippines, Hawaii and many parts of Africa [117–119]. Phytochemical study of the leaves of *M. oleifera*, resulted in the isolation of two new caffeoyl quinic acid glucosides (**109** and **110**), together with three known caffeoyl quinic acids, 3-*O*-, 4-*O*-, and 5-*O*-caffeoyl quinic acids, as well as five known flavonoid glucosides including quercetin 3-*O*-β-D-(6″-*O*-malonyl)-glucoside. The structures of new caffeoyl quinic acid glucosides were elucidated by spectroscopic analysis and chemical examinations to be 4-*O*-(4′-*O*-α-D-glucopyranosyl)- and 4-*O*-(3′-*O*-α-D-glucopyranosyl)-caffeoyl quinic acids (**109** and **110**, respectively) (Fig. 25) [120]. Glucosides of secondary metabolites found in plants normally have a β-configuration, since a glucosyl moiety is usually provided from UDP-glucose by SN2 nucleophilic displacement reaction, yielding a β-glucoside. In contrast, the α-glucosyl moiety is provided by ADP-glucose by SNi-like glucosyl transfer, and α-glucosides of secondary metabolites isolated from plants are few [121–124]. From this viewpoint, **109** and **110** are biogenetically interesting secondary metabolites with an α-linked glucopyranosyl residue. In an influenza A neuraminidase inhibitory assay, 3-*O*-caffeoyl- and 5-*O*-caffeoyl quinic acids displayed moderate inhibitory activity with IC_50_ values of 30.0 and 27.8 μg/mL, respectively, while 4-*O*-caffeoyl quinic acid was less potent (IC_50_ 67.0 μg/mL). Quercetin 3-*O*-β-D-(6"-*O*-malonyl)-glucoside also showed moderate influenza A neuraminidase inhibitory activity, with an IC_50_ value of 25.3 μg/mL.

## Conclusion

This review summarized phytochemical studies on traditional herbal medicines used in China, Mongolia, Uzbekistan, and Bangladesh, which were performed based on the ethnopharmacological information obtained by field studies or those exchanges collaborated with researchers of the respective regions. From traditional herbal medicines used in the regions above, together with a traditional herbal medicine related to one of those used in Hokkaido, diverse classes of new molecules were isolated, and their structures were elucidated on the basis of NMR, MS, and ECD analyses including a TDDFT ECD calculation method as well as chemical conversions. Our phytochemical studies on traditional herbal medicines uncovered structurally and biogenetically unique molecules.

In the studies on Chinese traditional herbal medicines especially used in Yunnan Province, and Guangxi Zhuang Autonomous Region, phytochemical studies of eight traditional herbal medicines, as well as one unexplored plant and one related herbal medicine used in Hokkaido, yielded several structurally unique molecules. Rigenolide A (**1**) isolated from *Gentiana rigescens* was a unique secoiridoid glucoside formed by intramolecular [2 + 2] cycloaddition, while rigenolides B (**7**) and C (**8**) were diastereomeric conjugates of a secoiridoid glucoside and a norsecoiridoid. Spicachlorantins C–F (**11**–**14**) isolated from *Chloranthus spicatus* were the first lindenane sesquiterpene dimers with a hydroperoxy group at C-4, being considered to be biogenetic precursors for the corresponding hydroxy derivatives of dimeric lindenane sesquiterpenes. From the same genus plant, *C. japonicus*, which has been used by an ethnic minority group living in Hokkaido, hitorins A (**16**) and B (**17**), novel C_25_ terpenoids with a 6/5/5/5/5/3 hexacyclic skeleton, being considered to be biogenetically derived from eudesmane sesquiterpene and thujane monoterpene, were isolated.

The information of medicinal uses for *Linaria buriatica* and *Lophanthus chinensis* were obtained in the ethnopharmacological survey of Mongolian traditional herbal medicines, and they were found to be phytochemically unexplored plants. Linaburiosides A‒D (**47**–**50**), iridoid glucosides possessing an iridoid derived acyl moiety, isolated from *Linaria buriatica*, whose iridoid derived acyl moiety were only found in *Linaria* plants. Algiolide A (**57**), isolated from a traditional herbal medicine, *Gentiana algida,* used in Mongolia, has a novel skeleton consisting of fused α,β-unsaturated δ-lactone and cyclopentene rings, being considered to be derived from gentiopicroside commonly exists in the Gentianaceous plants.

From *Perovskia scrophulariifolia*, an herbal medicine traditionally used in Uzbekistan, perovsfolins A (**71**) and B (**72**), possessing a C_28_ terpenoid moiety with unprecedented 6/8/6/6/6 pentacyclic carbon skeleton, were isolated, which were presumed to be produced by condensation of a 20-norabietane diterpene and methyl rosmarinate. Paeobrin (**87**) isolated from *Paeonia hybrida* possessed a new carbon framework, appeared to be derived from paeoniflorin as for lactiflorin and they were biogenetically related to each other, while paeonihybridin (**86**) had a hybrid structure consisted of paeoniflorin and paeonovicinoside. The first naturally occurring polyacetylen glucoside possessing an α-pyrone moiety (**81**) was isolated from *Mediasia macrophylla*.

In contrast, in an evaluation of biological activities for the isolated compounds, a variety of activities including neuroinflammatory activity, antiproliferative activities against human tumor cell lines as well as reversing activity MDR of tumor cells, antimicrobial activities against various bacteria and fungus, antiviral activities against influenza A virus, or influenza A neuraminidase inhibitory activity, and anti-allergic activity through suppression of H1R mRNA up-regulation useful for therapeutic agents, as well as proproliferative activity on human follicle dermal papilla cells beneficial for hair care were also demonstrated.

Ethnopharmacological surveys have provided useful information for performing phytochemical study to find a variety of new molecules with unique structures and various biological activities. Our study results suggested that there are still many traditional herbal medicines should be explored as resources of new molecules as seeds of therapeutic agents.
